# Hypoparathyroidism, Sensorineural deafness and renal disease (Barakat syndrome) caused by a reduced gene dosage in *GATA3*: a case report and review of literature

**DOI:** 10.1186/s12902-019-0438-4

**Published:** 2019-10-28

**Authors:** Anne D. D. Joseph, Nirmala D. Sirisena, Thirunavukarasu Kumanan, Vathualan Sujanitha, Veronika Strelow, Raina Yamamoto, Stefan Wieczorek, Vajira H. W. Dissanayake

**Affiliations:** 1 University Medical Unit, Teaching Hospital Jaffna, Jaffna, Sri Lanka; 2 Human Genetics Unit, Faculty of Medicine, University of Colombo, Colombo 8, Sri Lanka; 3 MVZ Dr. Eberhard & Partner Dortmund GbR (ÜBAG), 44137 Dortmund, Germany

**Keywords:** Barakat syndrome, *GATA3*, HDR syndrome, Hypocalcaemia, Hypoparathyroidism, Renal dysplasia, Sensorineural deafness

## Abstract

**Background:**

Barakat syndrome is an autosomal dominant rare genetic disease caused by haploinsufficiency of the GATA binding protein 3 (*GATA3*) gene. It is also known as HDR syndrome, and is characterized by varying degrees of hypoparathyroidism, sensorineural deafness and renal disease. This is the first report of a heterozygous *GATA3* whole gene deletion causing HDR syndrome in a Sri Lankan family.

**Case presentation:**

A 13-year-old boy with an acute febrile illness, hypocalcaemia and bilateral carpopedal spasm was referred for evaluation. A past medical history of treatment for persistent hypocalcaemic symptoms since the age of 7 months was obtained. Biochemical investigations showed persistent low serum corrected calcium levels with hyperphosphataemia, hypomagnesaemia, low parathyroid hormone levels, hypercalciuria, and low total 25-hydroxy vitamin D levels. His renal functions and renal sonography were normal. Audiometry showed bilateral moderate to severe sensorineural hearing loss. On screening, his mother was also found to have asymptomatic hypocalcaemia, hypomagnesaemia, hyperphosphataemia, hypercalciuria and low total 25-hydroxy vitamin D levels. She had impaired renal functions and chronic parenchymal changes in the renal scan. Audiometry showed bilateral profound sensorineural hearing loss. Genetic analysis using multiplex-ligation dependent probe amplification showed a reduced gene dosage for *GATA3* that is consistent with a heterozygous whole gene deletion in both the child and mother.

**Conclusions:**

This report demonstrates the wide intra-familial phenotypic variability observed in HDR syndrome and adds further to the existing scientific literature on the genotype-phenotype correlation of this syndrome. It highlights the need for HDR syndrome to be considered in the differential diagnosis of persistent hypocalcaemia with sensorineural deafness and/or renal involvement, and for appropriate genetic evaluation to be done to confirm the diagnosis.

## Background

Calcium homeostasis in the human body is finely regulated within a narrow physiological range and plays a vital role in maintaining cell stability and survival. It is mainly regulated through intestinal, osseous, and renal metabolism. Deficiency of calcium ions disturbs the integrity of the internal and external environment of cells. Hypoparathyroidism is a well-known cause for hypocalcaemia. Barakat syndrome, characterized by the triad of hypoparathyroidism, sensorineural deafness and renal disease, was first described in 1977 by Barakat et al. in 2 brothers with steroid-resistant nephrosis, nerve deafness, and hypoparathyroidism [1, 2]. It was named hypoparathyroidism, sensorineural deafness and renal disease (HDR) syndrome (OMIM#146255) by Hasegawa et al. [3]. This clinical entity is genetically heterogeneous and entails a wide spectrum of genotypic and phenotypic variations [3]. HDR syndrome is a rare autosomal dominant genetic disorder with variable expressivity and penetrance caused by haploinsufficiency of the GATA binding protein 3 (*GATA3*) gene (OMIM#131320) on chromosome 10p14 [4]. The *GATA3* gene consists of 6 exons that spans 20 kb of genomic DNA and encodes a 444-amino acid transcription factor with 2 transactivating domains (TA1, TA2) and 2 zinc finger domains (ZF1, ZF2) encoded by exons 2–6 [4]. GATA3 is one of 6 members of the GATA family of transcription factors that is involved in vertebrate embryonic development of the parathyroid glands, auditory system, kidneys, thymus and central nervous system. Studies have demonstrated the involvement of the GATA family of zinc finger transcription factors in the aetiology of several human malformations [4].

In HDR, hypoparathyroidism is characterized by either symptomatic or asymptomatic hypocalcemia along with undetectable or low serum levels of parathyroid hormone (PTH). The sensorineural deafness is usually bilateral, although the degree of hearing impairment is variable. Renal anomalies are also reported to be heterogeneous [5]. Herein, we describe the first report of a heterozygous *GATA3* whole gene deletion causing HDR syndrome in a Sri Lankan family.

## Case presentation

The proband is a 13-year-old boy who presented to the emergency unit with bilateral carpopedal spasm along with an acute febrile illness. He had a history of similar events since the age of 7 months, presumably triggered by febrile conditions due to respiratory tract infections. He had recurrent muscle cramps and lethargy associated with acute febrile illnesses, and in-between these episodes, he was apparently well. He is the third child of a non-consanguineous couple and was delivered by normal-vaginal delivery, with a birth weight of 2.5 kg. His developmental milestones were age-appropriate and immunization schedule was up-to-date. He has two elder siblings who are apparently healthy.

**Fig. 1:**
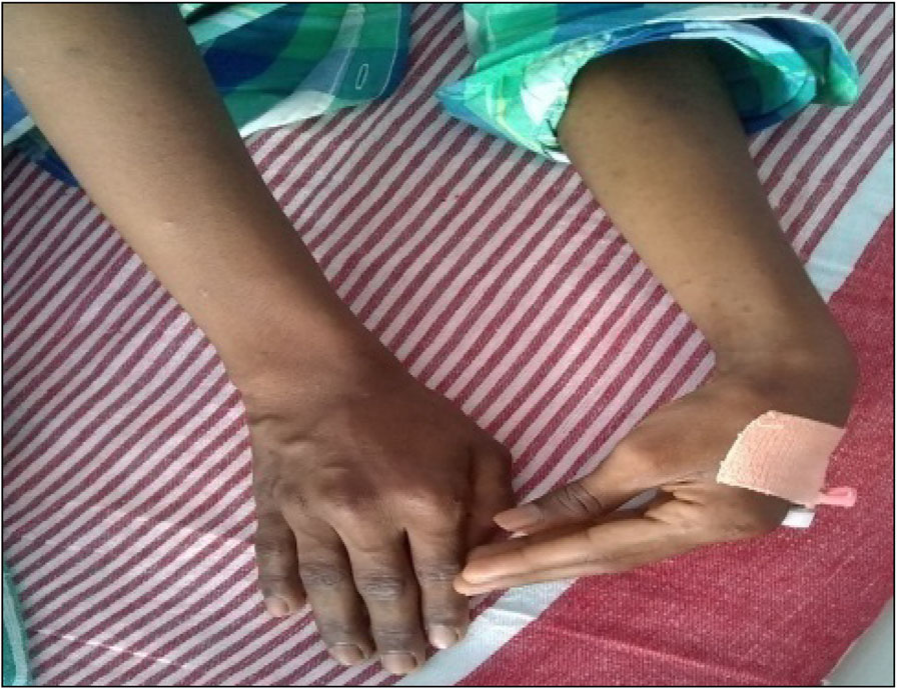
Carpopedal spasm in the proband with acute hypocalcaemia

On admission to the emergency unit, he was alert, conscious and febrile with stable vital signs. He had carpopedal spasm involving mainly the upper limbs (Fig. 1), which was reproducible by inflating a blood-pressure cuff placed on the patient’s arms. Chvostek’s sign was negative. He had diminished deep tendon reflexes in both upper and lower extremities with flexor plantar response. There was no papilledema, mental slowness or seizures. No facial dysmorphism was observed and other systemic examinations were unremarkable.

Blood samples were taken for basic investigations including full blood count and inflammatory markers, which were all normal. He had persistent low serum corrected calcium levels with hyperphosphatemia, mild hypomagnesaemia, and low parathyroid hormone levels. His renal functions, urine full report, arterial blood gas analysis and renal tract imaging were unremarkable. Other hormonal assays including Thyroid stimulating hormone (2.27 mIU/ L), free thyroxine (1.37 ng/dL), follicle stimulating hormone (2.5 mIU/L), luteinizing hormone (1.01 mIU/L), 9 am cortisol (312 nmol/L), prolactin (138.7 mIU/L) and testosterone (0.6 nmol/L) were within the normal range.

**Fig. 2:**
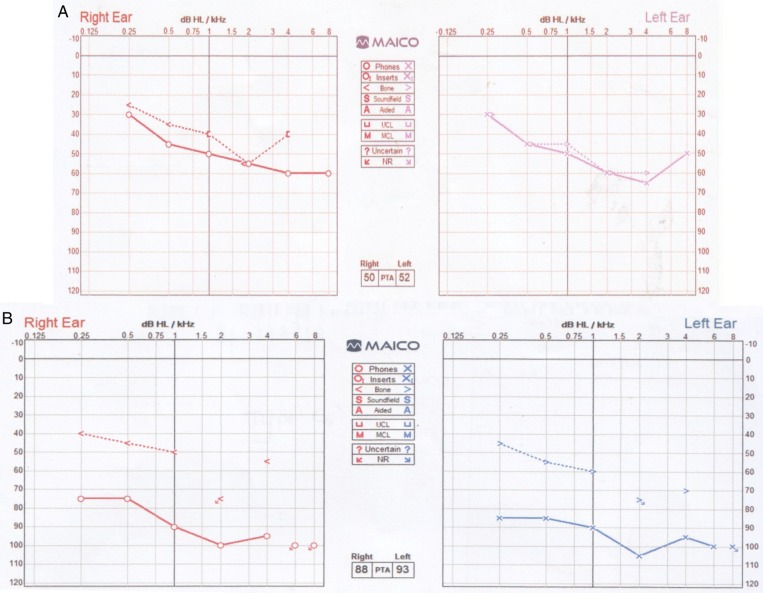
Audiometry findings in the proband (**a**) and the mother (**b**)

During the clinical interview, it was noticed that the mother of the child had some hearing impairment. Family screening was done in the parents and siblings with measurements of serum calcium, phosphate, magnesium and 24-h urinary calcium levels. The results showed that the mother of the boy also had similar biochemical findings. Laboratory investigations in the child and the mother are summarized in Table 1. Despite the hypocalcaemia, the mother was asymptomatic up to the age of 47 years. Interestingly, she was found to have chronic renal parenchymal disease and no renal dysplasia on imaging studies. Audiometry showed bilateral moderate sensorineural hearing impairment in the child and profound sensorineural hearing impairment in the mother, as shown in Fig. 2.

**Table 1:** Results of laboratory investigations in the proband and the mother

Test	Values in the child	Values in the mother	Reference range
Serum corrected calcium (mmol/L)	1.6	1.8	2.1–2.54
Serum phosphate (mmol/L)	2.71 mmol/L	1.91	0.18–1.45
Serum magnesium (mmol/L)	0.65 mmol/L	0.62	0.66–0.95
Parathyroid hormone (PTH) (pg/mL)	7.1 and 9.2	6.8	7.5–53.5
24-h urinary calcium (mmol/day)	1.348; adjusted 0.0385 mmoL/kg/day	3.360; adjusted 0.0412 mmoL/kg/day	normal up to 0.06 mmoL/kg/day
Total 25-hydroxy vitamin D (ng/mL)	25.4	15.1	30–100
Serum creatinine (μmol/L)	65	246	88–115
Blood urea (mmol/L)	4.6	11.2	2.5–7.1
Serum potassium (mmol/L)	4.0	4.8	3.5–4.5
Serum sodium (mmol/L)	137	140	135–147
ALP (IU/L)	150	68	44–147
Urine full report and cytology	Normal No active sediment	Normal No active sediment	
Renal sonography	Normal sized, symmetric kidneys	Bilateral chronic renal parenchymal disease	

*ALP* Alkaline phosphatase

**Fig. 3:**
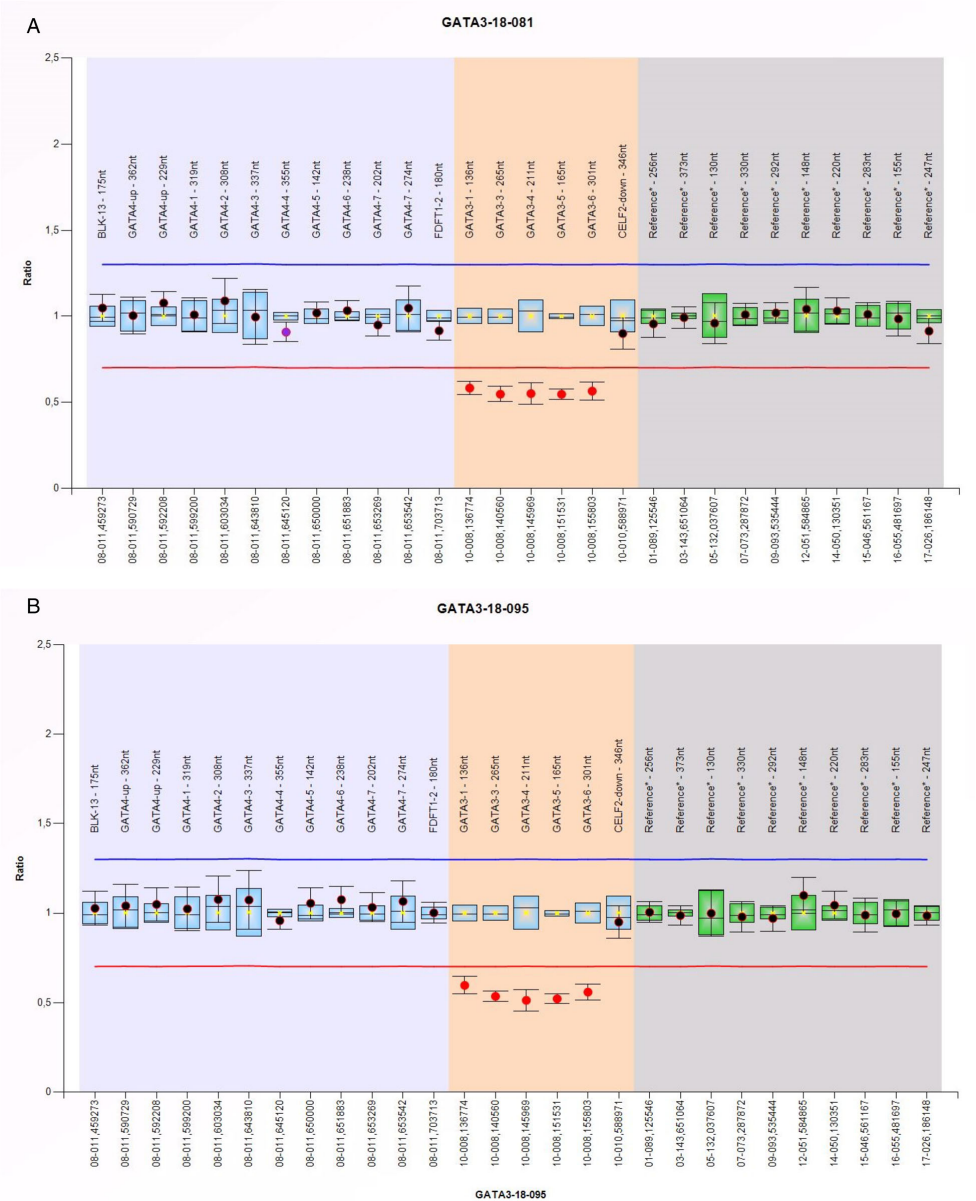
MLPA analysis in the proband (**a**) and mother (**b**) showing the *GATA3* whole gene deletion (reduced gene dosage/ratio for all *GATA3*-probes; sample was analyzed against 3 normal controls)

Because of the clinical findings, calcium-sensing receptor gene (*CASR*)-associated hypocalcemia (OMIM#601198) was initially suspected in the family. Sanger sequencing (exons 2–7 and flanking intronic sequences; NM_000388.3) and multiplex ligation-dependent probe amplification (MLPA; CASR P177-B2, MRC Holland) was performed but yielded normal results for the *CASR* gene. Unexpectedly, MLPA showed a reduced gene dosage for one single reference probe in the 10p14 genomic region specific for exon 6 of the *GATA3* gene. Therefore, further testing with MLPA kit P234-A3 (GATA3/4; MRC Holland; contains probes for exons 1 and 3–6 of *GATA3*) was performed and showed a reduced gene dosage for all *GATA3*-specific probes. The results of this MLPA analysis were consistent with a heterozygous whole gene deletion of *GATA3* (minimum size of the deletion 19 kb) in both the proband and his mother (Fig. 3). As the probe for the *CELF2* gene (approximately 2433 kb downstream of *GATA3*) showed a normal gene dosage, the downstream breakpoint of the deletion is localized between *GATA3* and *CELF2*. The deletion breakpoint upstream of *GATA3* could not be determined with the analyses performed.

After hospitalization, the child was administrated 10 mg of 10% calcium gluconate, intravenously over 10 min, 8-hourly. The child’s symptoms improved and the serum total calcium rose to 2.1 mmol/L. From the second day of hospitalization, the patient was started on oral calcium supplements 50 mg/kg/day and 1, 25-hydroxyvitamin D 0.5 μg/day. He was subsequently discharged on outpatient follow-up. His mother was treated similarly and maintained near normal levels of serum calcium and phosphorus. Both the child and his mother were referred for otolaryngology follow-up with a long-term plan for providing hearing aids. Additionally, the mother was referred to the nephrology team for follow-up of the renal impairment. Regular clinic follow-up with serum calcium levels and annual renal sonography was arranged for the proband.

## Discussion and conclusions

The HDR syndrome, also known as Barakat syndrome is as an autosomal dominant rare genetic disorder [2], primarily caused by haploinsufficiency of *GATA3* gene on chromosome 10p14 [4]. *GATA3* is expressed in the developing parathyroid glands, inner ears and kidneys, together with the thymus and central nervous system [5]. Genetic variations that can cause HDR syndrome include missense or nonsense pathogenic variants, small insertions or deletions and large deletions, which cause structural variations in the *GATA3* gene [5]. However, it is reported that identifiable *GATA3* variants are not present in all patients with clinical features compatible with the HDR syndrome [6]. In this family, both the proband and his mother had a heterozygous whole gene deletion of *GATA3*.

HDR syndrome is highly heterogeneous. The triad of hypoparathyroidism, sensorineural deafness and renal disease is usually observed in 62.3% of patients; 28.6% of patients show only hypoparathyroidism and deafness and 2.6% of patients present with only deafness and renal disease [7]. Hypoparathyroidism in HDR syndrome can range from asymptomatic to myalgia, neuromuscular irritability, non-febrile seizures or pronounced tetany caused by severe hypocalcemia. Hypoparathyroidism is known to have a variable age of onset and is characterized by symptomatic or asymptomatic hypocalcaemia with undetectable or low serum PTH levels. Renal anomalies in HDR syndrome are also highly heterogeneous and include renal dysplasia, hypoplasia, aplasia, cystic kidneys and vesicoureteral reflux [5]. Proteinuria, haematuria, renal tubular acidosis and nephrocalcinosis have also been reported [8]. However, most patients show progression to chronic renal failure and often require renal replacement therapy. The prognosis of patients affected with HDR syndrome generally depends on the severity of the renal disease.

Hearing impairment is the most consistent feature of the syndrome. Patients usually present with early onset, moderate to severe sensorineural hearing impairment which is mostly bilateral, symmetric and slightly worse at the higher end of the frequency spectrum [9]. The higher frequency sensorineural hearing impairment is known to progressively worsen with age [6, 7].

In this family, even though the child and mother had the same genetic defect, the phenotypic features were somewhat variable. This intra-familial phenotypic variation is a characteristic feature of the HDR syndrome. A similar wide-spectrum of phenotypic variation was described in other studies reported in the scientific literature [9–12]. The findings in the present case are compared with previously reported cases in Table 2.

**Table 2:** Summary of phenotypic and genotypic characteristics of the present case and previously reported cases with HDR syndrome

	Present case		Akie Nakamura et al. (2011) [13]					Nasrollah Maleki et al. (2013) [14]	Liu C, et al. (2015) [15]	Gül Yesiltepe Mutlu et al. (2015) [16]	Xue-Ying Chu et al. (2017) [17]	Tetsuji Okawa et al. (2015) [7]
	Proband	Mother	Patient 1	Patient 2	Patient 3	Patient 4	Patient 5					
Gender	Male	Female	Female	Female	Female	Female	Male	Male	Male	Male	Female	Female
Age at diagnosis	13 years	47 years	1 month	11 months	2 years	1 month	13 years	58 years	19 years	13 years	14 years	33 years
Parathyroid function at the time of diagnosis												
Hypoparathyroidism	Yes	Yes	Yes	Yes	Yes	Yes	Yes	Yes	Yes	Yes	Yes	Yes
Clinical features	Tetany	Asymptomatic	Poor weight gain	Seizures	Lower limb pain	Seizures	Muscle cramps	Seizures	Tetany	Tetany	Seizures	Seizures
Serum calcium (mmol/L)	1.6	1.85	1.2	1.1	unknown	1.5	1.4	1.325	1.86	1.675	1.62	1.3
Serum phosphate (mmol/L)	2.71	1.91	unknown	2.907	unknown	3.714	2.196	2.32	1.71	3.068	3.70	1.51
Intact PTH (pg/mL)	7.1	6.9	7–10	5–9	20	9	13	5	9.96	20	22	7
Sensorineural deafness	Moderate	Profound	+	+	+	+	+	Moderate to severe	+	+	+	+
Hearing level right/ left ears (dB)^a^	50/52	92/97	60/60	60/45	60/80–100	80/80	40–50/ 40–50	20–80/ 20–80	60–80/ 60–100	–	–	47/ 55
Age at diagnosis	13 years	47 years ^b^	2.8 years	8 years	11 years	–	–	–	2 years	–	11 years	41 years
Renal anomaly	Normal	CKD	Normal	Normal	Normal	Renal dysplasia	Normal	CKD	Small kidneys	Small pelvic left kidney	Normal	Right renal dysplasia
Abnormality in the *GATA3* gene	Heterozygous whole gene deletion of *GATA3* (min.19kbp)	Heterozygous whole gene deletion of *GATA3* (min.19kbp)	Exon 6 c.1063delC p.L355X	Exon 3 c.432insG p.K303X	Exon 4 c.784A > G p.R262G	Intron 5/exon 6 boundary c.1051-1G > T p.1351fsX18	Exon 5 c.942 T > A p.C318S	Not identified	Exon 2 c.529dupC p.Arg177profsX126	Exon 4 p.R276Q c.827G > A	Exon 2 c.286delT p.W96GfsX99	Exon 4 p.R299Q

^a^Degree of hearing loss: normal: < 25 dB; mild: 26–40 dB; moderate: 41–55 dB; moderately severe: 56–70 dB; and profound: > 90 dB; ^b^had undetected hearing loss since childhood; *CKD* chronic kidney disease, *PTH* parathyroid hormone

As denoted in the previous reports, hypoparathyroidism is a consistent and common feature. However, even though sensorineural deafness was also commonly reported, the definite time of its onset is not well known, as it is a slowly progressive disorder and early medical attention is not usually sought by most of the patients. At the time of the clinical evaluation, if the patient has profound or demonstrable deafness or there is a family history of deafness, this may provide a clue regarding the underlying HDR syndrome. If mild to moderate deafness is not identified during routine clinical examination and the patient also is unaware of its presence, the diagnosis often gets delayed. This is a gray area in this disease. As reported in the published literature, most of the HDR cases were initially managed mainly as primary hypoparathyroidism [13]. In the present case, the child was initially thought to suffer from *CASR*-related primary hypoparathyroidism since he had normal developmental milestones and average school performance, and the slowly progressive deafness was identified only later.

Renal anomalies in the HDR syndrome have a wide phenotypic variation and the age of onset is also variable. In the current case, the proband exhibited hypoparathyroidism and sensorineural deafness, but has not yet developed renal manifestations. The proband’s mother exhibited all three classical features of the HDR syndrome. When all three features are present or when patients have two features with a positive family history, HDR syndrome could easily be diagnosed. In such instances, considering the cost and availability of testing, genetic confirmation is often considered optional [6]. It is important to consider Barakat syndrome as a differential diagnosis in patients with isolated sensorineural deafness or renal impairment who have a family history of any of these conditions. In such patients, *GATA3* testing for confirmation of the diagnosis is indicated [6].

In conclusion, this study reports a heterozygous whole gene deletion of the *GATA3* gene responsible for the HDR syndrome in a Sri Lankan family with wide intra-familial phenotypic variability. This case emphasizes that in the evaluation of persistent hypocalcaemia with renal and/or sensorineural deafness, HDR syndrome should be considered. Comprehensive renal and audiometry assessments should be done in clinically suspected patients, to establish the diagnosis and to provide specific appropriate care and rehabilitation. *GATA3* genetic studies should be performed in every suspected patient and the family members should also be screened for hypoparathyroidism, deafness, and renal involvement. Additional genetic studies should be done where indicated to identify the precise molecular genetic defects in patients with the HDR syndrome in order to further elucidate the genotype-phenotype correlation of this rare syndrome.

## Data Availability

All data generated in this study are included in this published article.

## Abbreviations

ALP: Alkaline phosphatase
ALT: Alanine transaminase
AST: Aspartate transaminase
*CASR*: Calcium-sensing receptor gene
CRP: C-reactive protein
ESR: Erythrocyte sediment rate
*GATA3*: GATA binding protein 3
HDR: Hypoparathyroidism, sensorineural deafness and renal involvement
MCV: Mean corpuscular volume
MLPA: Multiplex ligation-dependent probe amplification
PTH: Parathyroid hormone
WBC: White blood cells
